# Genome Mining in Glass Chemistry Using Linear Component Analysis of Ion Conductivity Data

**DOI:** 10.1002/advs.202301435

**Published:** 2023-05-07

**Authors:** Zhiwen Pan, Jan Dellith, Lothar Wondraczek

**Affiliations:** ^1^ Otto Schott Institute of Materials Research University of Jena 07743 Jena Germany; ^2^ Leibniz Institute of Photonic Technologies – IPHT 07743 Jena Germany; ^3^ Center of Energy and Environmental Chemistry University of Jena 07743 Jena Germany

**Keywords:** genome mining, glass, ionic conductivity, materials genome, property predictions

## Abstract

Understanding the multivariate origin of physical properties is particularly complex for polyionic glasses. As a concept, the term genome has been used to describe the entirety of structure‐property relations in solid materials, based on functional genes acting as descriptors for a particular property, for example, for input in regression analysis or other machine‐learning tools. Here, the genes of ionic conductivity in polyionic sodium‐conducting glasses are presented as fictive chemical entities with a characteristic stoichiometry, derived from strong linear component analysis (SLCA) of a uniquely consistent dataset. SLCA is based on a twofold optimization problem that maximizes the quality of linear regression between a property (here: ionic conductivity) and champion candidates from all possible combinations of elements. Family trees and matrix rotation analysis are subsequently used to filter for essential elemental combinations, and from their characteristic mean composition, the essential genes. These genes reveal the intrinsic relationships within the multivariate input data. While they do not require a structural representation in real space, how possible structural interpretations agree with intuitive understanding of structural entities known from spectroscopic experiments is finally demonstrated.

## Graphical abstract

Understanding the multivariate origin of physical properties is particularly complex for polyionic glasses. Here, the genes of ionic conductivity are derived from strong linear component analysis of a uniquely consistent dataset, and are presented as fictive chemical entities. Their possible structural interpretations are demonstrated to agree with spectroscopic experiments.

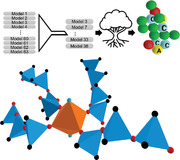

## 1 Introduction

Relations between chemical composition, the spatial arrangement of atoms (structure), and the evolving macroscopic properties are of central interest in glass science and technology.^[^ ^1^, ^2^, ^3^, ^4^, ^5^, ^6^, ^7^ ^]^ The concentration of a single chemical element (or nominal compound) is the intuitive descriptor for starting to explore these relations. However, this simplistic approach is usually insufficient to account for material behavior in glasses containing multiple chemical elements, in particular, for properties as complex as ionic conductivity.^[^ ^3^, ^8^, ^9^, ^10^ ^]^ A plot of a material property over the concentration of a single element may show, in this case, broadly scattered data, a vertical line, or some kind of mathematical trend with many or only a few outliers, all of which can be highly misleading. Instead, such observations might be caused by insufficient expressivity when using single‐component concentration values as descriptors: multi‐component glasses usually exhibit intricate, typically unknown non‐linear relationships and interdependencies involving multiple elemental species. From this, the immediate question arises as to how multi‐component descriptors can be identified for a given glass property, i.e., the glass genome.^[^ ^11^ ^]^ Are all or only some of the elements forming the material involved in individual such descriptors? What is the molar ratio of each element in these descriptor constructions? Can the descriptors be traced back – directly or indirectly – to structural entities or building blocks known to exist in the material? In the following, we will demonstrate how these questions can be answered through analyzing composition – property correlations for strong linear components, introducing Strong Linear Component Analysis (SLCA) for deciphering glass property genomes on small datasets.

**Figure 1:**
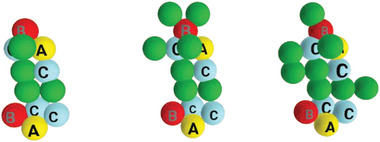
Schematic of the gene ABC_2_ as the common feature in three different functional groups responsible for a particular glass property. Green spheres represent unnamed elemental entities, e.g., A, B, C, or D.

In this context, the term gene is used for functional groups of elements acting as descriptors for a particular property. Genes are presented as fictive chemical entities with a characteristic stoichiometry derived from SLCA. They do not need to be charge‐balanced, and may not even require that their constituents are chemically connected, thus, they do not require a structural representation in real space (although such knowledge could be helpful in their further interpretation). As an example, three functional groups which could be responsible for ion conductivity are depicted schematically in **Figure** **1**. All of these groups involve the gene ABC_2_ as a common feature, whereas further constituents (depicted as green balls) could be any of the three elements A, B and C, or even additional, unknown components. In this schematic example, the gene of ABC_2_ would have been extracted by SLCA as a fundamental descriptor for ionic conductivity in the specific type of glass.

Technically, the present method is based on an optimization problem that maximizes the *R* ^2^ from linear regression^[^ ^12^ ^]^ between a property (here: ionic conductivity) and the sum of weighted concentrations of each constituent by tuning the weighting factors. This optimization is done for all subsets of composition – property data (that is, for all possible combinations of elements; each combination is handled as an individual model). Assisted by matrix rotation, principal component analysis (PCA,^[^ ^13^ ^]^) and family trees, those models that are essential for the studied property are identified, and from their characteristic mean composition, the essential genes. Thereby, the absolute concentration of a gene is initially undetermined; other than with common regression analysis or similar machine learning approaches which are frequently applied in glass science,^[^ ^14^ ^]^ relationships between the weighted sum of certain elemental components and a target property are not in the focus of the present study. The genes, however, may act as descriptors in physics‐informed ML models for glass property predictions.

We base our analysis on a uniquely consistent set of 56 glasses from the Na_2_O‐P_2_O_5_‐AlF_3_‐SO_3_ family, fabricated according to a uniform protocol, with exact compositional data and property measurements available from experimental study.

## 2 Results and Discussion

### 2.1 PCA and Dataset Dimensionality

The significance values *λ* after applying PCA on chemical composition data are listed in **Table** **1**. By summing up the significance values of the principle components PC1‐PC3, we find that three PCs are sufficient to explain 99.80% of the variations within the dataset. When only PC1 and PC2 are used, this value reduces to 90.62%. Thus, using more than three variables (genes) will probably overfit the current dataset, while using only two variables leads to underfitting. This reduction in dataset dimensionality (from 6 to 3) can be understood intuitively from the correlation between anion and cation fractions (charge compensation), mass conservation, and possible systematic correlations produced by interrelated batching (e.g., when using Na_2_SO_4_ or AlF_3_ for batching S or F).

**Table 1:** PCA of the six‐dimensional Na_2_O‐P_2_O_5_‐AlF_3_‐SO_3_ glass dataset. ***λ*** is presented as the percentage of the sum of all eigenvalues; it indicates the relevance (in terms of underlying property variance) of each component in property predictions

$\tilde{v}$	PC1	PC2	PC3	PC4	PC5	PC6
***λ*** (%)	70.44	20.17	9.18	0.19	0.01	0.00
PC1 + PC2 + PC3 + PC4				99.99		
PC1 + PC2 + PC3				99.80		
PC1 + PC2				90.62		

### 2.2 *R* ^2^ Optimization

The optimized *R* ^2^ value describes the best linear relationship found in each model by optimizing the weighting vector ***w***. The set of obtained values is provided in Table S1, Supporting Information. Results obtained for conductivity at different temperatures show very similar trends, therefore, we will initially limit the discussion to conductivity at 50 °C. The overall results in Table S1, Supporting Information show that the *R* ^2^ value increases from group I (*d* = 1) to group VI (*d* = 6); for *d* = 6 (involving all six elements into the SLCA procedure), a maximum of *R* ^2^ = 0.919 is obtained. For increasing temperature, there is a slight decrease in the optimized *R* ^2^ values. In order to indicate the descriptive power of each model, the individual *R* ^2^ values in Table S1, Supporting Information are stated relative to the maxima found for *d* = 6; in the following, we will refer to these relative values.

The group I models (including only one single elemental species) have *R* ^2^ ranging from 23.2% to 72.2%; only when correlating conductivity to P, a value of 92% is obtained (whereby we assume that this is an artifact originating from the narrow range of phosphate concentrations involved in the dataset, and further from interrelated batching). A significant improvement is observed in the group II models, for which the *R* ^2^ values increase up to 99.2%. This reflects the PCA, where an increase in confidence from ≈70% to ≈90% was seen when moving from one single PC to two PCs. In Group III, the linearity further improves slightly, with most models achieving *R* ^2^ above 99.2%. Groups IV, V, and VI show no more significant improvement in the relative *R* ^2^ value, with almost all models hitting 100%. Although we are not optimizing for the maximum variance such as in the PCA, group‐to‐group comparison strongly resembles the results of PCA. This is because the total sum of squares in the calculation of the *R* ^2^ value is proportional to the variance of data. Finally, the decreasing *R* ^2^ with increasing temperature indicates that the conductivity depends less on composition for higher temperatures.

### 2.3 Weighting Vectors and Mean Compositions

**Figure 2:**
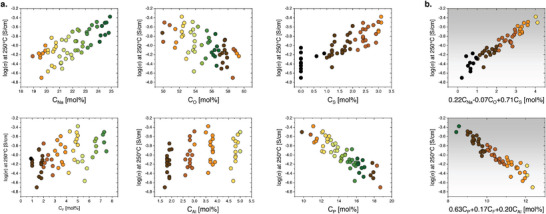
a) Correlations between experimental ionic conductivity at 250 °C and single‐element concentrations. b) Rescaled correlations to weighted composition using the [Na/O/S] and [P/F/Al] genes (see Table 2 for gene parameters).

The weighting vectors *w{w_1_, …., w_d_}*_i_ (corresponding to the optimized *R* ^2^ in each model) are used to calculate the chemical formulas of conductivity genes. Optimized such weighting vectors with *R* ^2^ > 99.0% (group III; *d* = 3) are listed in **Table** **2**. Mean compositions ***c*** are then calculated using Equation (8), multiplying ***w*** with the mean concentration $\bar{X}$ of the individual elements in Table S2, Supporting Information. The components in ***c*** are normalized to the quantity of the element following the order of priority O>Na>P>Al>F>S (ascending with the ratio Std./Mean in Table S2). The most stable element is always used as a reference in normalization. The elements Na, O, and P are relatively more stable than that S, F, and Al. The signs of components in ***c*** are forced positive. In this way, ***c*** represents the stoichiometry of genes underlying the present dataset. The practical consequence of this procedure is illustrated by way of example in **Figure** **2**, showing the correlation between experimental conductivity and the concentration of single elemental components (Figure 2a). Alternatively, strongly enhanced linearity is achieved when correlating to the weighted sum of [Na/S/O] using ***w*** = [0.22/0.71/‐0.07] or [P/F/Al] with *w* = [‐0.63/‐0.17/‐0.20] (see Figure 2b; *w* from Table 2). The corresponding mean composition ***c*** from these two models using $\tilde{X}$ = [Na/S/O] and $\tilde{X}$ = [P/F/Al] are [0.47/0.15/1] and [1/0.04/0.06]; these represent the genes $Na_{0.47}S_{0.15}\bar{O}$ and $\bar{P}{\bar{F}}_{0.04}{\bar{Al}}_{0.06}$, respectively (the macron on the elemental symbol representing a negative sign, that is, inverse proportionality). The absolute magnitude of the weighted sum concentrations can be scaled arbitrarily without changing the *R* ^2^ values. This is why the absolute concentration of the gene is undetermined. The standard deviation represents the uncertainty of gene stoichiometry, which we express in the general composition of each gene (see Table 2).

**Table 2:** Essential genes with their mean stoichiometry and weight vectors ***w***, general compositions and *R* ^2^ values. Signs on the weight factors are chosen so that positive factors signify increasing conductivity. The components of the weight vector are listed in the same order as in the model $\tilde{X}$ representation. Data are provided for conductivity at 50, 150, and 250 °C

essential gene	gene (*σ*_50°C_)	general composition (*σ*_50°C_)	R^2^ (*σ*_50°C_)	gene (*σ*_150°C_)	R^2^ (*σ*_150°C_)	gene (*σ*_250°C_)	R^2^ (*σ*_250°C_)
**Gene 1** **[Na/O/S]**	$Na_{0.47}\;S_{0.15}\bar{O}$ **0.17:0.70:‐0.13**	$Na_{0.47}\;S_{0.09-0.25}\bar{O}$	**0.913**	$Na_{0.66}\;S_{0.20}\bar{O}$ **0.17:0.72:‐0.10**	**0.901**	$Na_{1.20}\;S_{0.29}\bar{O}$ **0.22:0.71:‐0.07**	**0.895**
**Gene 2** **[Na/P/S]**	$Na\bar{P}S_{0.19}$ **0.20:‐0.30:0.50**	$Na\bar{P}S_{0.11-0.30}$	**0.914**	$Na{\bar{P}}_{0.74}S_{0.22}$ **0.20:‐0.22:0.58**	**0.903**	$Na{\bar{P}}_{0.41}\;S_{0.19}$ **0.24:‐0.15:0.61**	**0.896**
**Gene 3** **[Na/S/F]**	***Na*** ***S***_0.22_ ***F***_0.11_ **0.22:0.65:0.13**	***Na*** ***S***_0.13 − 0.35_ ***F***_0.06 − 0.17_	**0.912**	***Na*** ***S***_0.23_ ***F***_0.08_ **0.22:0.68:0.10**	**0.900**	***Na*** ***S***_0.20_ ***F***_0.05_ **0.25:0.68:0.07**	**0.894**
**Gene 4** **[Na/S/Al]**	***Na*** ***S***_0.19_ ***Al***_0.12_ **0.23:0.58:0.19**	***Na*** ***S***_0.11 − 0.30_ ***Al***_0.08 − 0.16_	**0.915**	***Na*** ***S***_0.20_ ***Al***_0.10_ **0.23:0.62:0.16**	**0.904**	***Na*** ***S***_0.19_ ***Al***_0.07_ **0.25:0.63:0.12**	**0.897**
**Gene 5** **[Na/O/P]**	$Na_{0.33}{\bar{P}}_{0.79}O$ **0.17:‐0.62:0.21**	$Na_{0.33}{\bar{P}}_{0.70-0.90}O$	**0.913**	$Na_{0.29}{\bar{P}}_{0.67}O$ **0.17:‐0.59:0.23**	**0.904**	$Na_{0.33}{\bar{P}}_{0.59}O$ **0.21:‐0.55:0.24**	**0.897**
**Gene 6** **[P/F/Al]**	$\bar{P}{\bar{F}}_{0.04}{\bar{\mathrm{Al}}}_{0.06}$ **‐0.68:‐0.12:‐0.20**	$\bar{P}{\bar{F}}_{0.02-0.06}{\bar{\mathrm{Al}}}_{0.04-0.08}$	**0.913**	$\bar{P}{\bar{F}}_{0.08}{\bar{\mathrm{Al}}}_{0.04}$ **‐0.68:‐0.21:‐0.12**	**0.904**	$\bar{P}{\bar{F}}_{0.07}{\bar{\mathrm{Al}}}_{0.07}$ **‐0.63:‐0.17:‐0.20**	**0.896**

### 2.4 Determination of Essential Genes

**Figure 3:**
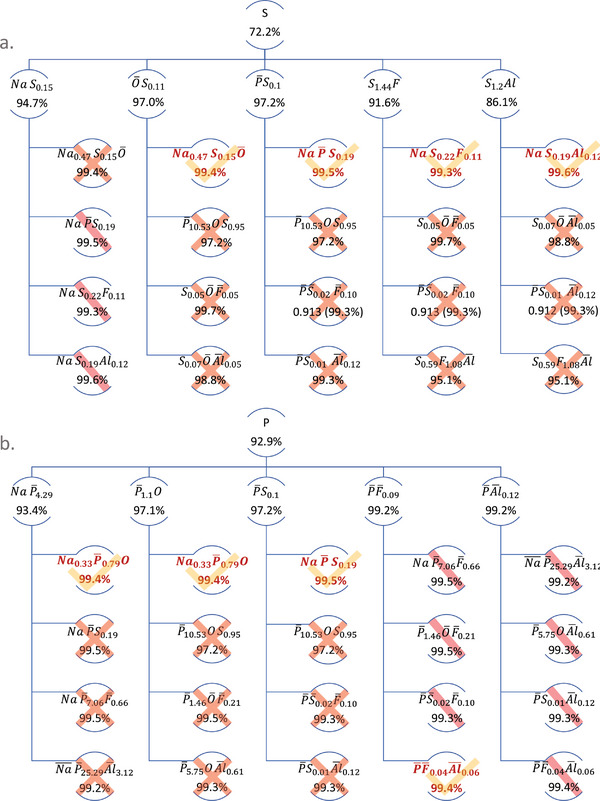
Family trees originating from [S] a) and [P] b). The essential genes in group III are highlighted; overfit models are crossed out (for uncertain models, a bar instead of a cross is used). Numbers indicate the relative *R* ^2^ value obtained for each model.

As a crucial step in the SLCA, the set of models needs to be filtered for essential genes which represent neither underfitted nor overfitted models. From the PCA, we know that the hyperparameter *n_d_* should not exceed 3, otherwise, the model is overfitted. However, this information alone is not sufficient to identify the essential models. The *R* ^2^ values in group III still vary from 54.0% to 99.8%, meaning that some models are underfitted while others are overfitted. Removing the models with low *R* ^2^ to avoid underfitting within group III is straightforward. Excluding overfitted models is less obvious. Using *R* ^2^ alone cannot distinguish between over‐fitted and just well‐fitted models. Therefore, an additional criterion is applied on the models of group III, whereby a model is considered overfitted when *R* ^2^ is high, but not rooted in group hierarchy: adding one more element from group to group must improve model linearity. **Figure** **3** shows two families originating from S and P, selected by way of example for their highest *R* ^2^ among the group I models. On the second level of the family trees, only those models (group II) are considered descendant from S (or P) which (i) include S (or P) and (ii) have higher *R* ^2^ than their parent. On the third and any higher level, in addition to (i‐ii), the molar ratio and qualitative contribution (signified by a positive or negative sign) of the parent elements must also reflect in the child's model stoichiometry (iii). For example, all of the five possible group II descendants of S have increased *R* ^2^ values, therefore, they are all children of S. Each of these five children has four further descendants in group III, however, the majority of those second‐level descendants is excluded because of the stoichiometry criterion (iii), or because of an insignificant increase in *R* ^2^ over the group II ancestor (indicating overfitting). As a result, we identify four essential genes, and a limited number of uncertain models (for which exclusion based on deviating stoichiometry is less clear; the main reason for such uncertainty is with the limited size of the dataset relative to the strong variation in the elemental concentration of *S*).

The same procedure is carried out for element P and its group II and group III descendants. Here, models [P/F] and [P/Al] achieved a relative *R* ^2^ of 99.2% already in group II; for symmetry reasons, they both converge in group III [P/F/Al] with a slight further increase in *R* ^2^. Overall, two additional, unique genes are identified in this way, whereas the [Na/P/S] model recurred in both the S and the P families. The six essential genes are summarized in Table 2.

When conductivity is correlated to glass composition using the gene weighting factors, highly refined linear correlations are obtained, see Figure 2b; the composition of the essential genes represents the internal correlation among the chemical components in determining ionic conductivity. In Figure 2, the essential models [Na/S/O] and [P/F/Al] are used for this demonstration. They do not contain any common element and, consequently, seem to conjugate in their contribution to ionic conductivity.

Five of the six essential genes involve Na; expectedly, it is the most important element in increasing the ionic conductivity. Beyond Na, S is the second most frequent species, therefore, we will focus our discussion on the structural meaning of the identified genes on these two elements. In addition, we find that the Na contribution within increases consistently with temperature, in each of the genes in which it is present. We will therefore close the following discussion with a perspective on the temperature‐dependence of Na mobility.

### 2.5 Structural Significance of Essential Genes

**Figure 4:**
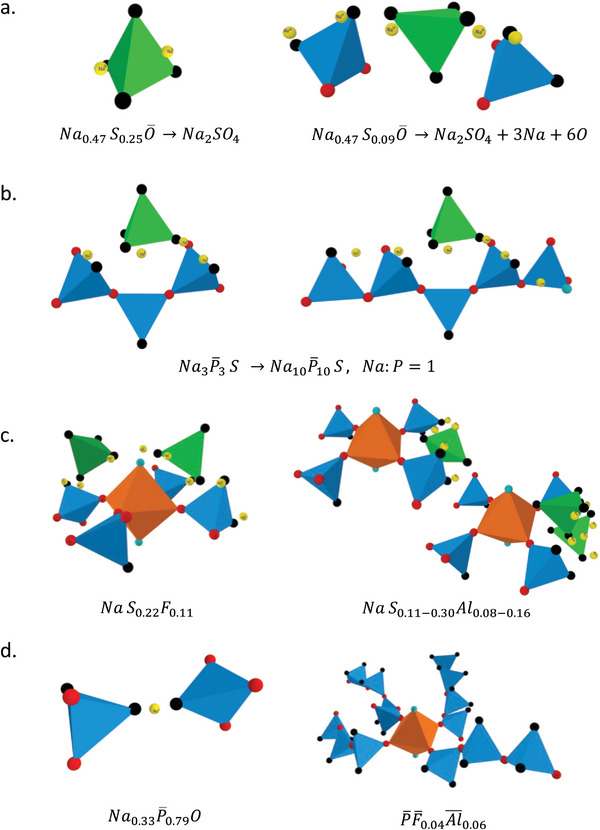
Schematic representations of possible structural interpretations of essential genes for ionic conductivity in Na_2_O‐P_2_O_5_‐AlF_3_‐SO_3_ glasses. Balls represent chemical species: Na (yellow), F (turquoise), bridging oxygen (red) and non‐bridging oxygen (black). The green tetrahedron depicts and SO_4_ group; the orange octahedron is Al(O,F)_6_.

Combining gene composition and bonding information can potentially reveal the structural significance of the identified genes. In order to explore this aspect, we first interpret each element as a structural entity:^[^ ^6^, ^7^, ^8^, ^16^ ^]^ S as a sulfate tetrahedron, P as a phosphate tetrahedron, Al as an alumina octahedron, F as a terminal P‐F or Al‐F bond, or as an Al‐F‐Al link, and Na as Na^+^ ions attached to F terminal bonds, non‐bridging oxygen species, or isolated sulfate tetrahedra. Assumed charge carriers are Na(+)^[^ ^17^, ^18^ ^]^ and, eventually, F(‐) and O(2‐). We now consider each gene separately, using the general stoichiometry (taking into account the standard deviation found per gene for the contributions of S, F, and Al; the gene itself is specific, however, uncertainty arises from the underlying dataset). In **Figure** **4**, possible structures are depicted for each gene at the extremes of its respective general stoichiometry.

(1) $$Na_{0.47}\;S_{0.09-0.25}\bar{O}$$

On the upper end of its general formula, the [Na/S/O] gene involves two Na+ ions per one SO_4_^2−^ tetrahedron, that is, a sodium sulfate group (Figure 4a, left). Lower S signifies an excess of Na^+^, charge compensated by non‐bridging oxygen species (i.e., up to 3 Na and 6 O when S is at its minimum value of 0.09). A possible structural representation of this is depicted in Figure 4a (right), where a sulfate tetrahedron is surrounded by the additional oxygen species in a mix of bridging and nonbridging configurations between network‐forming polyhedra, and charge‐compensating the additional Na^+^ ions. In effect and although the exact chemical environment is uncertain, the structural role of the [Na/S/O] gene, therefore, is that of an SO_4_ bridge in an Na‐rich environment. This agrees with the observation of the conjugate [P/F/Al] gene discussed in the previous section. The bulk conductivity then depends on the frequency of these ionic bridging entities, which – assumedly – would interact to form ion transport channels.

(2) $$Na\bar{P}S_{0.11-0.30}$$

The normalized extreme cases of the [Na/P/S] gene are $Na_{3}{\bar{P}}_{3}S$ and $Na_{10}{\bar{P}}_{10}$. Both formulas maintain a fixed Na:P ratio of 1. This stoichiometry reflects the interaction between a network former (P) and a network modifier (Na), bridged by sulfate entities. It involves phosphate groups with 3 to 10 tetrahedra per sulfate ion. Compared to the [Na/S/O] gene, [Na/P/S] is more specific in terms of representing similar sulfate bridges, but now within a defined range of superstructural network arrangements (Figure 4b).

(3) $$NaS_{0.13-0.35}F_{0.06-0.17}\;and\;NaS_{0.11-0.30}Al_{0.08-0.16}$$

In [Na/S/F] and [Na/S/Al], the phosphate group is replaced by F and Al, respectively. Again, both genes represent SO_4_ bridging units, however, at the ‐P‐F‐Al‐ phosphate junctions. The S:F ratio in [Na/S/F] is fixed at 2:1, indicating that a sulfate bridge involves two sulfate tetrahedra and one terminal F (Figure 4c, left). The [Na/S/Al] gene has an S:Al ratio varying from 2:1 to 1:1, meaning that in this case, the sulfate bridge consists of one or two sulfate tetrahedra (Figure 4c, right).

(4) $$Na_{0.33}{\bar{P}}_{0.79}O\;and\;\bar{P}{\bar{F}}_{0.04}{\bar{\mathrm{Al}}}_{0.06}$$

In normalized form, the gene of [Na/P/O] is $Na{\bar{P}}_{2.5}O_{3}$; it represents a simple sodium phosphate in which conductivity increases with increasing sodium concentration, and decreases for higher phosphate content (Figure 4d, left). The interesting aspect is with the contribution of O: in contrast to the role of bridging oxygen in the above [Na/S/O] gene, in the non‐bridging form, gene mining accurately predicts that oxygen increases conductivity. Finally, the [P/F/Al] gene is the conjugate of [Na/S/O], as discussed in the previous paragraphs. It represents a phosphate network cross‐linked by ‐Al‐ O – and –Al‐F‐ entities (Figure 4d, right).

### 2.6 Temperature Effects

The SLCA was carried‐out for conductivity data collected at 50, 150, and 250 °C (Table 2). The [Na/P/O] and [P/F/Al] genes are practically insensitive to temperature within this range, indicating structural stability below *T*_g_. For all other genes and, in particular, [Na/O/S], the relative contribution of Na increases notably with increasing temperature. At the same time, the weight of P in [Na/P/S] decreases. These observations reflect that temperature affects primarily the sulfate bridge in its strength to localize Na ion species. With increasing temperature, the sulfate entity interacts with less and less phosphate groups and, in turn, assembles more Na in its vicinity. Overall, it is not surprising that the gene stoichiometry is temperature dependent, given the multitude of interaction potentials within multi‐component glasses such as the present ones.

## 3 Conclusion

In the context of the material genome, genes are functional groups of elements acting as descriptors for a particular property. Studying ionic conductivity in glasses from the Na_2_O‐P_2_O_5_‐AlF_3_‐SO_3_ family, we presented them as six fictive chemical entities with a characteristic stoichiometry derived from strong linear component analysis. SLCA maximizes the quality of linear regression between a property (here: ionic conductivity) and champion compositions from all possible combinations of elements. Family trees and matrix rotation allow for the identification of essential genes, which are filtered from the set of all possible combinations of elements present in the considered dataset.

While such genes do not require a structural representation in real space, we finally demonstrated how possible structural interpretations agree with intuitive understanding of structural entities known from spectroscopic experiments.

## 4 Experimental Section

### General

**Figure 5:**
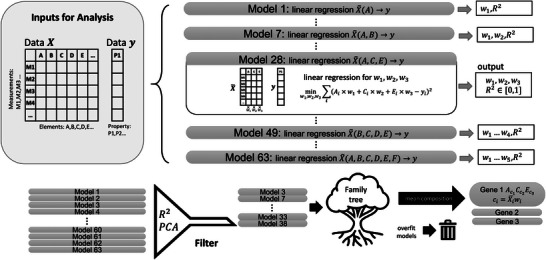
Schematic of the gene extraction method using SLCA. SLCA stars with a linear regression and optimization for maximum *R* ^2^ of composition – property correlations for all possible combinations of elements, based on the weighted sum of elemental concentrations (leading to 63 individual models). Optimized weighting factors and *R* ^2^ values are then used to construct family trees, and models are filtered by PCA and physical ancestry. From this, essential models are obtained, whose mean compositions are referred to as essential genes.

A general schematic of the approach used to extract genes for glass conductivity is shown in **Figure** **5**. We initially constructed 63 linear regression models according to all possible subsets $\tilde{X}$ of independent variables (elemental constituents) in the input matrix *X* (Weight Vector Optimization). The choice of linear models reflects Occam's razor, but it is not unique: other model orders could have been chosen for higher complexity. A weighted sum of the independent variables in $\tilde{X}$ is generated in each linear regression model, yielding a total *R* ^2^ feedback value as a function of the individual weighting factors used in the model for each variable. By tuning the weighting factors, *R* ^2^ is maximized for a particular model; the optimized weighting factors and the optimized *R* ^2^ are the output values of this procedure. All optimized models are subsequently categorized into groups according to the number of independent variables in $\tilde{X}$. PCA is used to filter for groups that are neither underfit nor overfit (Principal Component Analysis). Family trees are then constructed, in which models are probed for ancestry by number of components, and unphysical models are removed. The remaining models are referred to as *essential models*, and their mean compositions are the essential genes.

### Dataset

As proof of principle, we focus on gene extraction for the (sodium) ion mobility in glasses of the Na_2_O‐P_2_O_5_‐AlF_3_‐SO_3_ family. For these glasses, a highly consistent dataset including 56 individual samples with variable chemical composition is available from our lab, together with a range of physical and spectroscopic data.^[^ ^6^, ^7^, ^8^ ^]^ In order to improve the accuracy of chemical composition data over previously available energy‐dispersive X‐ray spectroscopic results (EDX),^[^ ^15^ ^]^ all glasses were reanalyzed by fully quantitative wavelength‐dispersive X‐ray spectroscopy (WDX) using a JEOL JXA8800L microprobe analyzer. Generally, compared to standardless EDX the WDX technique provides improved energy resolution, detection limit, and more precise results, in particular, for the lighter elements such as F (the elemental composition obtained in this way and the previously reported ionic conductivity are the (X, Y) input in Figure 5).

All samples were coated with a layer of approximately 5 nm of carbon before WDX measurement to avoid charging effects under the electron beam. In order to minimize beam damage of the sensitive Na_2_O‐P_2_O_5_‐AlF_3_‐SO_3_ glasses the count rate of the elements in question was initially recorded as a function of beam time, and the excitation conditions were optimized accordingly. As a result, the energy of the exciting electron was set to *E*_0_ = 10 kV, and a moderate beam current of 30 nA was applied. The beam diameter was defocused to 100 µm. As diffractive element a TAP crystal (2*d*: 25.757 Å) was used for the detection of the KL_3_ radiation of F (677 eV), Na (1040 eV), and Al (1486 eV), and a PET crystal (2*d*: 8.742 Å) for P (2010 eV) and S (2309 eV). All lines were recorded over a time of 60 s (peak) and 2 × 30 s (background), respectively. These times were chosen to guarantee sample stability under the electron beam; for observation times of 120 s, we started to see dynamic variations in the Na ion concentration.

For each glass, the SLCA was carried‐out three times, using conductivity data at three different temperatures (50, 150, and 250 °C) so as to obtain information on the possible effect of temperature on gene prevalence. The full dataset is provided in the supplementary Table S2.

### Weight Vector Optimization

Vector optimization was conducted using linear fits of the composition – conductivity relationship to obtain weight factors for the contribution of individual elements to ionic conductivity. For an *n*‐dimensional (number of elemental constituents) multivariate dataset ***X*** with *m* observations (number of composition datasets), and another *m* × 1 dataset y with *m* observations (number of ionic conductivity datapoints), the SLCA searches for the optimal *d* × 1 vector ***w*** = [*w*_1_,*w*_2_…*w_d_*]^*T*^ (gene receipt) such that

(5) $$R^{2}=1-\frac{\sum_{i\;=\;1}^{m}{\left(y_{i}-f_{i}\right)}^{2}}{\sum_{i\;=\;1}^{m}{\left(y_{i}-\bar{y}\right)}^{2}}$$

is maximized (thereby, *d* is the vector dimensionality, 1 < *d* < 6). The *R* ^2^ value ranges from 0 to 1, representing poor (*R* ^2^ → 0) and perfect linearity (*R* ^2^ = 1). *y_i_* and $\bar{y}$ are the *i*th observation and mean value of *y*, respectively. *f_i_* is the predicted value from the best linear fit between the *m* × 1 vector ***x*** = ***Xw*** and the *m* × 1 vector ***y***. ***x*** is the weighted sum of elements in ***X***, with weights ***w***.

(6) $$f\;\left(w\right)=ax+b=a\mathrm{Xw}+b$$

where *a* and *b* are the slope and intercept obtained via minimizing the least square error, respectively,

(7a) $$\min_{a,b}\sum_{i\;=\;1}^{m}{\left(f_{i}-y_{i}\right)}^{2}$$

(7b) $$\max_{w}\left(1-\frac{\sum_{i\;=\;1}^{m}{\left(y_{i}-f_{i}\left(w\right)\right)}^{2}}{\sum_{i\;=\;1}^{m}{\left(y_{i}-\bar{y}\right)}^{2}}\right)$$

There are two optimization processes in SLCA. One is to minimize the least square error (Equation (7a)) in order to obtain the slope and intercept for the best linear fit, and the other one is to obtain the weighting factor ***w*** by maximizing the *R* ^2^ value based on Equation (7b). Since ***w*** is a *d* × 1 vector, we have *d* − 1 fitting parameters (because of normalization, $|\sum_{i\;=\;1}^{m}w_{i}|$ = 1, therefore, |*w*_1_| = 1 when *d* = 1). The remaining fitting parameters are initiated 1000 times randomly between the lower and upper bounds of ‐1 and 1, respectively. The two optimization problems form one single model (Figure 5) with ***w*** and *R* ^2^ as output. SLCA than explores all possible subsets $\tilde{X}$ of ***X*** without requiring any filtering procedure (from prior knowledge) before data input. In the present case, six elements are considered, *n* = 6. This results in $C_{6}^{1}+C_{6}^{2}+C_{6}^{3}+C_{6}^{4}+C_{6}^{5}+C_{6}^{6}=6+15+20+15+6+1=63$ possible elemental combinations (with the subscript *n*, and the superscript *d*, the number of constituents partaking in the specific model; e.g., $C_{6}^{1}$ could represent a model correlating all data to Na concentration alone, whereas $C_{6}^{6}$ is the model in which all elements are considered in the fitting process). Each such combination represents one individual model for which the above optimization is done using the full dataset of 56 samples. The weight vector ***w*** therefore varies in its dimensionality *d*, corresponding to the model dimensionality of $\tilde{X}$. In the following, the models will be grouped according to their dimensionality, from group I to group VI.

The individual weighting factors *w_i_* in the vector ***w*** represent the contribution of element *i* to the target property, here, ionic conductivity. The sign of *w_i_* is defined by forcing the slope *a* in Equation (6) to be positive, which is equivalent to requiring monotonically increasing proportionality for the element *i* with positive *w_i_* and vice versa.

The weighting factors alone are not suitable for representing genes in terms of (fictive) chemical formulas because they are not given in molar ratios. Therefore, we transform to ***c*** instead, with

(8) $$c_{i}=w_{i}\;\bar{X_{i}}$$

where $\bar{X_{i}}$ is the mean of the *i*th chemical element in subset $\tilde{X}$. In this way, genes are represented in the form of ***c***.

### Principal Component analysis

PCA is used to analyze the entire dataset in order to reveal its minimum dimensionality. The principal components ***v*** of the *m* × *n* dataset ***X*** are obtained by solving the eigenvalue equation

(9) Cv=λv

where *λ*_*i*_ is the *i*th eigenvalue of the corresponding eigenvector ***v***_***i***_ (*i*th principal component). ***C*** is a *n* × *n* matrix, the covariance matrix:

(10) $$C=\left[\begin{array}{ccc} Cov\left(X_{1},X_{1}\right) & \;\cdots & Cov\left(X_{1},X_{n}\right)\\ \vdots & \;\ddots & \;\vdots \\ Cov\left(X_{n},X_{1}\right) & \;\cdots & \;Cov\left(X_{n},X_{n}\right) \end{array}\right]$$

where *Cov*(*X_i_*,*X_j_*) is the covariance of two variables *X_i_* and *X_j_*, which are the *i*th and *j*th element/column of ***X***:

(11) $$Cov\;\left(X_{i},X_{j}\right)=E\left(\left(X_{i}-\bar{X_{i}}\right)\left(X_{j}-\bar{X_{j}}\right)\right)$$

where *E* is the expected value operator, and $\bar{X_{i}}$ and $\bar{X_{i}}$ are mean of variables *X_i_* and *X_j_*, respectively. The PCA initially generates the same number of principal components as there are dimensions in the input dataset. It then reduces dimensionality by removing those components which have low *λ*_*i*_. The remainder is the principal components; their number is the minimum dimension of the dataset. In effect, PCA conducts a matrix rotation aiming to identify correlations in the raw dataset which would reduce its dimensionality.

Before conducting PCA, the raw dataset ***X*** requires standardization,

(12) $$Z_{i}=\frac{\left(X_{i}-\bar{X_{i}}\right)}{E\left({\left(X_{i}-\bar{X_{i}}\right)}^{2}\right)}$$

The standardized *Z_i_* (centered at 0 and scaled by forcing its variation to 1) now replaces *X_i_* in Equations ((5), (9))–(11) so that the PCA is not biased in any chemical element *i* with high absolute magnitude in *X_i_*. To keep the coefficients in principal components ***v*** orthonormal, the components *v_i_* in ***v*** are rescaled by the standard deviation of the corresponding *X_i_*,

(13) $$\tilde{v}=\mathrm{Dv}=\left[\begin{array}{ccc} \frac{1}{Std\left(X_{1}\right)} & \;\cdots & 0\\ \vdots & \;\ddots & \;\vdots \\ 0 & \;\cdots & \;\frac{1}{Std\left(X_{n}\right)} \end{array}\right]\;v$$

where ***D*** is the *n* × *n* diagonal matrix with *n* diagonal elements equal to the inverse of standard deviations of *n* variables/column, *X*_1_, *X*_2_…*and* *X_n_*. Finally, the orthonormal $\tilde{v}$ are obtained (shown in Table 1 as PC1, PC2 … PC6, following in ascending order their Eigenvalues *λ*_1_ > *λ*_2_ > *λ*_3_ > *λ*_4_ > *λ*_5_ > *λ*_6_). For example, the first principal component for our nominal data is (with T representing the transpose)

(14) $${\tilde{v}}_{1}={\left[-0.33,+0.48,+0.46,-0.17,-0.46,-0.46\right]}^{T}$$

## Conflict of Interest

The authors declare no conflict of interest.

## Supporting information

Supporting Information

## Data Availability

The data that support the findings of this study are available in the supplementary material of this article.
