# Supplementary material for: Genome Mining in Glass Chemistry Using Linear Component Analysis of Ion Conductivity Data
Source: Adv Sci (Weinh). 2023 May 7;10(21):2301435. doi: 10.1002/advs.202301435 (PMC10375087; doi:10.1002/advs.202301435)
Supplement: Supplementary file 1 — Supporting Information [file ADVS-10-2301435-s001.pdf]

ADVANCED SCIENCE
Open Access

Supporting Information

for *Adv. Sci.*, DOI 10.1002/advs.202301435

Genome Mining in Glass Chemistry Using Linear Component Analysis of Ion Conductivity Data

*Zhiwen Pan, Jan Dellith and Lothar Wondraczek**

Genome mining in glass chemistry using linear component analysis of ion conductivity data

Zhiwen Pan[1], *Jan Dellith*[2], *Lothar Wondraczek*[1,3]

[1]Otto Schott Institute of Materials Research, University of Jena, 07743 Jena, Germany

[2]Leibniz Institute of Photonic Technologies – IPHT, 07743 Jena, Germany

[3]Center of Energy and Environmental Chemistry, University of Jena, 07743 Jena, Germany

E-mail: lothar.wondraczek@uni-jena.de

Supplementary Information

Table S1. R^2 for the optimized linear contributions to conductivities σ at different temperatures, obtained using SLCA. Maximum values are found for group VI (d = 6); all other values are given relative to these maxima to indicate the descriptive power of each model.

	Model $\tilde{X}$	R^2 $\sigma_{50℃}$	R^2 $\sigma_{150℃}$	R^2 $\sigma_{250℃}$		**Model** $\tilde{X}$	R^2 $\sigma_{50℃}$	R^2 $\sigma_{150℃}$	R^2 $\sigma_{250℃}$
**Group I**	[Na]	60.4%	55.1%	59.6%		[O/P/F]	99.5%	100.0%	99.5%
	[O]	46.6%	42.4%	37.5%		[O/P/Al]	99.3%	99.5%	98.7%
	[P]	92.9%	87.9%	86.7%		[O/S/F]	99.7%	99.7%	99.4%
	[S]	72.2%	75.5%	76.7%		[O/S/Al]	98.8%	98.9%	97.8%
	[F]	34.9%	29.4%	25.5%		[O/F/Al]	54.0%	48.9%	49.4%
	[Al]	23.2%	19.1%	15.4%		[P/S/F]	99.3%	100.0%	99.3%
**Group II**	[Na/O]	62.4%	57.9%	59.8%		[P/S/Al]	99.3%	99.4%	98.7%
	[Na/P]	93.4%	89.3%	87.7%		[P/F/Al]	99.4%	99.9%	99.1%
	[Na/S]	94.7%	94.0%	97.6%		[S/F/Al]	95.1%	94.6%	93.0%
	[Na/F]	63.6%	59.7%	60.2%	**Group IV**	[Na/O/P/S]	99.5%	100.0%	99.3%
	[Na/Al]	64.8%	61.5%	60.9%		[Na/O/P/F]	99.6%	100.0%	99.7%
	[O/P]	97.1%	95.2%	94.8%		[Na/O/P/Al]	99.4%	100.0%	99.3%
	[O/S]	97.0%	97.6%	94.3%		[Na/O/S/F]	100.0%	100.0%	100.0%
	[O/F]	53.9%	48.6%	49.1%		[Na/O/S/Al]	99.7%	100.0%	99.6%
	[O/Al]	52.4%	47.0%	46.5%		[Na/O/F/Al]	95.6%	100.0%	95.4%
	[P/S]	97.2%	96.7%	94.6%		[Na/P/S/F]	99.5%	100.0%	99.4%
	[P/F]	99.2%	99.8%	98.8%		[Na/P/S/Al]	100.0%	100.0%	100.0%
	[P/Al]	99.2%	99.0%	98.6%		[Na/P/F/Al]	99.5%	100.0%	99.5%

	[S/F]	91.6%	91.6%	88.8%		[Na/S/F/Al]	99.7%	100.0%	99.7%
	[S/Al]	86.1%	86.8%	84.5%		[O/P/S/F]	99.7%	100.0%	99.6%
	[F/Al]	44.9%	38.0%	37.2%		[O/P/S/Al]	99.3%	100.0%	98.9%
Group III	[Na/O/P]	99.4%	99.9%	99.3%		[O/P/F/Al]	99.5%	100.0%	99.5%
	[Na/O/S]	99.4%	99.5%	99.0%		[O/S/F/Al]	99.9%	100.0%	99.9%
	[Na/O/F]	86.9%	89.1%	84.7%		[P/S/F/Al]	99.4%	100.0%	99.4%
	[Na/O/Al]	78.4%	80.4%	78.4%	Group V	[Na/O/P/S/F]	100.0%	100.0%	100.0%
	[Na/P/S]	99.5%	99.7%	99.1%		[Na/O/P/S/Al]	100.0%	100.0%	100.0%
	[Na/P/F]	99.5%	100.0%	99.4%		[Na/O/P/F/Al]	100.0%	100.0%	100.0%
	[Na/P/Al]	99.2%	99.2%	98.6%		[Na/O/S/F/Al]	100.0%	100.0%	100.0%
	[Na/S/F]	99.3%	99.4%	98.9%		[Na/P/S/F/Al]	100.0%	100.0%	100.0%
	[Na/S/Al]	99.6%	99.9%	99.2%		[O/P/S/F/Al]	100.0%	100.0%	100.0%
	[Na/F/Al]	67.5%	65.0%	65.4%	G. VI	[Na/O/P/S/F/Al]	100.0%	100.0%	100.0%
	[O/P/S]	97.2%	98.1%	94.8%		**Maximum**	0.919	0.906	0.904

Table S2. Chemical composition obtained by WDX data (data **X** in **Figure 5**) and conductivity σ dataset (data **y** in **Figure 5**) at different temperatures for 56 compositions in the Na_2O-P_2O_5-AlF_3-SO_3 glass system.

No.	*Na mol%*	*O mol%*	*P mol%*	*S mol%*	*F mol%*	*Al mol%*	*log (σ) 50°C S/cm*	*log (σ) 150°C S/cm*	*log (σ) 250°C S/cm*
1	22.26	53.92	13.62	1.76	4.60	3.84	-7.64	-5.36	-3.91
2	22.49	55.44	13.07	2.96	3.22	2.84	-7.42	-5.15	-3.68
3	24.17	52.49	13.38	1.28	4.96	3.72	-7.37	-5.20	-3.78
4	23.12	53.42	12.73	2.30	4.76	3.67	-7.28	-5.17	-3.71
5	24.32	52.48	12.49	1.96	4.96	3.80	-7.07	-4.92	-3.58
6	22.20	55.68	13.85	2.43	3.04	2.80	-7.69	-5.36	-3.91
7	22.43	53.71	14.34	1.17	4.69	3.66	-7.89	-5.56	-4.10
8	24.90	52.31	11.10	3.08	5.02	3.59	-6.87	-4.78	-3.38
9	24.47	52.63	12.10	2.44	4.85	3.51	-6.99	-4.88	-3.51
10	24.48	53.83	13.34	1.84	3.52	2.99	-7.63	-5.36	-3.77

11	23.93	54.30	12.92	2.56	3.53	2.75	-7.21	-5.03	-3.59
12	23.42	54.68	14.73	1.24	3.24	2.69	-8.01	-5.58	-3.99
13	21.64	57.26	15.95	1.58	1.76	1.82	-8.20	-5.79	-4.16
14	20.60	57.88	16.83	1.16	1.63	1.90	-8.28	-5.95	-4.47
15	21.20	57.68	15.46	2.08	1.59	1.99	-7.73	-5.38	-3.88
16	19.51	57.92	16.87	1.01	2.04	2.65	-8.50	-5.86	-4.26
17	20.96	56.44	15.87	1.13	2.75	2.84	-8.26	-5.82	-4.24
18	18.93	59.31	18.29	0.65	1.03	1.81	-8.43	-5.86	-4.23
19	19.92	58.91	16.73	1.60	0.96	1.88	-8.16	-5.67	-4.11
20	21.34	56.35	15.17	1.60	2.68	2.85	-8.00	-5.62	-4.10
21	19.72	57.78	16.22	1.39	2.07	2.81	-8.16	-5.73	-4.17
22	21.45	57.61	14.94	2.48	1.59	1.94	-7.89	-5.49	-3.89
23	20.57	58.67	16.10	1.93	0.87	1.84	-7.99	-5.63	-4.08
24	20.71	55.54	13.12	2.88	3.94	3.81	-7.71	-5.42	-3.94
25	20.40	55.72	13.87	2.37	3.86	3.77	-7.56	-5.31	-3.83
26	19.54	56.19	15.68	1.10	3.66	3.83	-8.30	-5.89	-4.21
27	20.67	53.84	13.53	1.75	5.44	4.77	-7.63	-5.40	-4.02
28	19.91	57.54	14.65	2.60	2.40	2.90	-7.84	-5.50	-4.00
29	20.11	55.99	15.02	1.56	3.64	3.70	-8.04	-5.69	-4.15
30	19.35	59.11	17.76	0.95	1.03	1.80	-8.30	-5.79	-4.17
31	20.53	53.88	14.19	1.24	5.41	4.75	-7.94	-5.62	-4.14
32	20.09	57.58	15.24	2.20	2.25	2.64	-8.07	-5.66	-4.10
33	24.13	49.97	12.13	1.29	7.50	4.97	-7.66	-5.40	-3.92
34	24.64	50.10	9.84	3.10	7.30	5.02	-7.08	-4.91	-3.50
35	23.89	53.68	12.18	2.98	4.28	2.98	-7.06	-4.90	-3.49
36	24.80	49.89	11.22	1.90	7.28	4.91	-7.41	-5.18	-3.73
37	21.66	52.34	12.19	2.39	6.62	4.79	-7.42	-5.23	-3.81

38	22.03	52.33	11.41	2.96	6.50	4.77	-7.30	-5.12	-3.70
39	23.52	50.84	10.76	2.79	7.16	4.94	-7.09	-4.95	-3.56
40	22.78	51.62	12.30	1.89	6.65	4.77	-7.60	-5.38	-3.93
41	23.30	51.45	11.49	2.44	6.61	4.71	-7.20	-5.04	-3.65
42	21.47	52.89	15.26	0.00	5.79	4.59	-8.24	-5.85	-4.29
43	23.19	51.45	14.33	0.01	6.27	4.75	-7.82	-5.55	-4.05
44	19.61	58.57	18.75	0.01	1.36	1.70	-8.66	-6.30	-4.70
45	21.94	57.02	16.19	1.21	1.76	1.89	-8.18	-5.69	-4.20
46	22.72	53.06	15.35	0.01	5.00	3.87	-8.03	-5.65	-4.15
47	20.36	56.63	17.56	0.01	2.82	2.63	-8.64	-6.10	-4.44
48	20.11	53.82	15.83	0.00	5.58	4.65	-8.71	-6.19	-4.56
49	22.23	56.81	15.50	1.70	1.90	1.85	-8.04	-5.62	-4.11
50	22.75	56.66	14.38	2.50	1.87	1.83	-7.73	-5.43	-4.02
51	22.42	53.71	12.72	2.46	4.85	3.83	-7.17	-5.09	-3.65
52	23.12	56.36	13.64	2.95	2.03	1.90	-7.53	-5.20	-3.75
53	21.06	54.77	16.34	0.00	4.18	3.64	-8.52	-6.04	-4.39
54	21.98	55.26	16.63	0.01	3.26	2.86	-8.35	-5.88	-4.28
55	21.13	57.32	17.94	0.01	1.76	1.84	-8.63	-6.06	-4.37
56	21.20	57.20	17.89	0.00	1.84	1.86	-8.78	-6.43	-4.50

	**Na**	**O**	**P**	**S**	**F**	**Al**
Mean	21.75	55.23	14.63	1.60	3.61	3.17
Std./Mean	7%	4%	13%	60%	49%	33%
